# Primary and secondary prevention of stroke and systemic embolism with rivaroxaban in patients with non-valvular atrial fibrillation: Sub-analysis of the EXPAND Study

**DOI:** 10.1007/s00380-018-1219-0

**Published:** 2018-07-06

**Authors:** Shinichiro Uchiyama, Hirotsugu Atarashi, Hiroshi Inoue, Takanari Kitazono, Takeshi Yamashita, Wataru Shimizu, Takanori Ikeda, Masahiro Kamouchi, Koichi Kaikita, Koji Fukuda, Hideki Origasa, Hiroaki Shimokawa

**Affiliations:** 1 Center for Brain and Cerebral Vessels, Sanno Hospital and Sanno Medical Center, International University of Health and Welfare, 8-5-35 Akasaka, Minato-ku, Tokyo, 107-0052 Japan; 2 Minamihachioji Hospital, 3-18-12 Koyasu-cho, Hachioji, Tokyo, 192-0904 Japan; 3 Saiseikai Toyama Hospital, 33-1 Kusunoki, Toyama, Toyama 931-8533 Japan; 4 Department of Medicine and Clinical Science, Graduate School of Medical Sciences, Kyushu University, 3-1-1 Maidashi, Higashi-ku, Fukuoka, Fukuoka 812-8582 Japan; 5 Cardiovascular Institute Hospital, 3-2-19 Nishiazabu, Minato-Ku, Tokyo, 106-0031 Japan; 6 Department of Cardiovascular Medicine, Graduate School of Medicine, Nippon Medical School, 1-1-5 Sendagi, Bunkyo-ku, Tokyo, 113-8602 Japan; 7 Department of Cardiovascular Medicine, Toho University Faculty of Medicine, 5-21-16 Omorinishi, Ota-ku, Tokyo, 143-8540 Japan; 8 Department of Health Care Administration and Management, Center for Cohort Study, Kyushu University Graduate School of Medical Sciences, 3-1-1 Maidashi, Higashi-ku, Fukuoka, Fukuoka 812-8582 Japan; 9 Department of Cardiovascular Medicine, Graduate School of Medical Sciences, Kumamoto University, 2-39-1 Kurokami Chuo-ku, Kumamoto, Kumamoto 860-8555 Japan; 10 Division of Heart Rhythm, International University of Health and Welfare Hospital, International University of Health and Welfare, 537-3 Iguchi, Nasushiobara, Tochigi, 537-3 Japan; 11 Division of Biostatistics and Clinical Epidemiology, University of Toyama Graduate School of Medicine, 2630 Sugitani, Toyama, Toyama 930-0194 Japan; 12 Department of Cardiovascular Medicine, Tohoku University Graduate School of Medicine, 1-1 Seiryomachi, Aoba-ku, Sendai, Miyagi 980-8574 Japan

**Keywords:** Non-valvular atrial fibrillation, Anticoagulation, Rivaroxaban, Stroke, Secondary prevention

## Abstract

The EXPAND Study examined the real-world efficacy and safety of rivaroxaban for the prevention of stroke and systemic embolism (SE) in Japanese patients with non-valvular atrial fibrillation (NVAF). In this sub-analysis, we compared the differences in efficacy and safety between patients with and those without history of stroke or transient ischemic attack (TIA). This multicenter, prospective, non-interventional, observational, cohort study was conducted at 684 medical centers in Japan. A total of 7141 NVAF patients aged ≥ 20 years [mean age 71.6 ± 9.4 (SD) years] who were being or planned to be treated with rivaroxaban (10 mg/day, 43.5%; 15 mg/day, 56.5%) were followed for a mean period of 897.1 ± 206.8 days with a high follow-up rate (99.7%). The primary prevention group comprised patients without history of ischemic stroke or TIA (*n *= 5546, 77.7%), and the secondary prevention group comprised those with history of ischemic stroke or TIA (*n *= 1595, 22.3%). In the primary and secondary prevention groups, the incidence rate of stroke or SE (primary efficacy endpoint) was 0.7 and 2.2%/year, respectively (*P *< 0.001), and the incidence rate of major bleeding (primary safety endpoint) was 1.2 and 1.5%/year, respectively (*P *= 0.132). For major bleeding events, the incidence rate of intracranial bleeding was 0.4 and 0.8%/year (*P *= 0.002) in the primary and secondary prevention groups, respectively. This sub-analysis of the EXPAND Study showed that the Japan-specific dosages of rivaroxaban were effective and safe in Japanese NVAF patients with and those without ischemic stroke or TIA in routine clinical practice.

**Electronic supplementary material:**

The online version of this article (10.1007/s00380-018-1219-0) contains supplementary material, which is available to authorized users.

## Introduction

The morbidity of stroke has been reported as 2.6% for adult population and 15.1% for elderly aged 65 years and above [1]. The elderly patients aged 75 years or above with atrial fibrillation (AF) have relatively higher risk of stroke and death [2, 3]. The risk of stroke in these patients was not different between Japan and UK [3]. As the morbidity rate continues to increase, medical expenses for treatment of stroke are also expected to increase [1]. The risk factors for stroke in patients with AF include those used to calculate the CHA_2_DS_2_-VASc score proposed in the European Society of Cardiology Guidelines in 2010 [4]. Among these risk factors, stroke/transient ischemic attack (TIA)/thromboembolism and elderly age (> 75 years) are associated with the highest risk [4]. History of stroke is a particularly strong risk factor for stroke, 2.5 times higher susceptibility to stroke development in affected patients [5]. In the previous study for secondary prevention of stroke, recurrent stroke continued to account for 25–30% of all stroke [6].

Rivaroxaban, one of the non-vitamin K antagonist oral anticoagulants (NOACs), was approved in Japan in April 2012 [7]. The efficacy and safety of rivaroxaban were demonstrated in the ROCKET AF Trial [8], and the safety of Japan-specific rivaroxaban dosages [15 mg/day in patients with creatinine clearance (CrCl) ≥ 50 ml/min; 10 mg/day in patients with CrCl 30–49 ml/min] was shown in the J-ROCKET AF Trial [9]. In the J-ROCKET AF Trial, evidence for the safety and efficacy in Japanese patients was not sufficiently accumulated because of the lack of data for patients with CHADS_2_ scores of 0 or 1 [9]. However, the recent findings of our EXPAND Study reported in 2017 and 2018 showed the efficacy and safety of rivaroxaban in routine clinical practice including patients with such scores [10, 11]. The present report describes a sub-analysis of our EXPAND Study, addressing the primary and secondary prevention of stroke and systemic embolism (SE) with rivaroxaban in patients with non-valvular atrial fibrillation (NVAF). The primary and secondary prevention groups were defined as patients with and without history of stroke or TIA, respectively. We expect that our sub-analysis will provide the demographic characteristics by primary and secondary prevention groups and ensure the safety and efficacy of rivaroxaban of Japan-specific dosages in routine clinical practice.

## Methods

### Research overview

The study design and the results of the main statistical analysis of the EXPAND Study were described previously [10, 11]. Briefly, the study was a multicenter, prospective, non-interventional, observational cohort study to demonstrate the efficacy and safety of rivaroxaban in patients with NVAF. Among patients aged ≥ 20 years with NVAF using or planned to use rivaroxaban who provided written consent, those not meeting the exclusion criteria were enrolled [10, 11]. Patients newly treated with rivaroxaban were defined as new users, and those already using rivaroxaban before providing informed consent were defined as current users [11].

The secondary and primary prevention groups comprised patients with and those without history of ischemic stroke or TIA. The primary efficacy endpoint was the composite cumulative incidence of symptomatic stroke (ischemic or hemorrhagic) and SE, and the primary safety endpoint was the cumulative incidence of major bleeding events [10, 11]. Major bleeding, defined using the International Society on Thrombosis and Haemostasis (ISTH) criteria, was reported by the participating physicians. The secondary safety endpoint was non-major bleeding events, including clinically relevant non-major bleeding. Events reported during the observation period were adjudicated for inclusion or exclusion by a clinical events in a committee independent of the research organization [10, 11].

### Statistical analysis

We tabulated the demographics by prevention group and performed a Chi square test for each factor. The analysis set for efficacy and safety included patients with any follow-up information available after providing informed consent. The cumulative incidence rates of the efficacy and safety endpoints (%/year) from the time of starting rivaroxaban to the initial onset of events, together with Kaplan–Meier estimates, were calculated by primary and secondary prevention groups to perform a log-rank test. All statistical analyses were performed using SAS software (SAS for Windows Release ver. 9.2 or later; SAS Institute Inc., Cary, NC).

### Governance

The EXPAND Study was conducted in accordance with the principles of the Declaration of Helsinki, the Ethical Guidelines for Clinical Studies from the Japanese Ministry of Health, Labour and Welfare, and all applicable laws and regulations in Japan. The protocol was reviewed and approved by the Institutional Review Boards and/or Ethics Committee at all of the participating study sites. All patients provided written informed consent before enrollment. The study was registered with Clinical trials.gov (number NCT02147444) and with the University Hospital Medical Information Network clinical trials registry (number UMIN000009376).

## Results

### Demographics of subjects enrolled by prevention group (efficacy and safety populations)

The number of centers participating in this study was 684, and 7178 patients were registered during the enrollment period from November 20, 2012 to June 30, 2014. The target number of subjects was 7166, where 7141 patients were evaluated during the observation period up to March 31, 2016. The mean follow-up period was 897.1 ± 206.8 days (median 918.0), and 25 patients were lost to follow-up (follow-up rate 99.7%) [10, 11].

Table 1 shows the demographic characteristics of the patients examined in the sub-analysis. The primary and secondary prevention groups contained 5546 (77.7%) and 1595 (22.3%) patients, respectively. The following factors were noted more frequently in the secondary prevention group compared with the primary prevention group; male sex, mean age, elderly (75 years old and above), CHADS_2_, CHA_2_DS_2_-VASc and HAS-BLED scores, peripheral artery disease, diabetes mellitus, dyslipidemia, history of bleeding/bleeding tendency, non-paroxysmal (persistent/permanent) AF, and use of concomitant antiplatelet drugs. Meanwhile, the following factors were noted less frequently in the secondary prevention group compared with the primary prevention group; mean body weight, mean CrCl, congestive heart failure and liver dysfunction. In secondary prevention group, the distribution of patient with history of ischemic stroke and TIA was 90.3% (*n* = 1440) and 13.7% (*n* = 219), respectively.

**Table 1:** Demographic and baseline clinical characteristics of patients according to primary and secondary prevention groups for stroke and systemic embolism (efficacy and safety populations)

	Overall (*n* = 7141)	Primary prevention group (*n* = 5546)	Secondary prevention group (*n* = 1595)	*P* value
Sex, male, *n* (%)	4838 (67.7)	3708 (66.9)	1130 (70.8)	0.003
Age, years, mean (SD)	71.6 (9.4)	70.9 (9.5)	73.9 (8.4)	< 0.001
Age ≥ 75, years, *n* (%)	2919 (40.9)	2093 (37.7)	826 (51.8)	< 0.001
Body weight, kg, mean (SD)	62.8 (12.5)	63.2 (12.6)	61.2 (12.1)	< 0.001
Creatinine clearance, ml/min, mean (SD)	69.7 (26.2)	71.2 (26.8)	64.7 (23.2)	< 0.001
< 30 ml/min, *n* (%)	133 (2.0)	104 (2.0)	29 (1.9)	< 0.001
30–49 ml/min, *n* (%)	1347 (19.8)	968 (18.3)	379 (24.9)	
≥ 50 ml/min, *n* (%)	5326 (78.3)	4213 (79.7)	1113 (73.2)	
CHADS_2_ score, mean (SD)	2.1 (1.3)	1.6 (1.0)	3.7 (1.0)	< 0.001
CHA_2_DS_2_-VASc score, mean (SD)	3.4 (1.7)	2.9 (1.4)	5.1 (1.4)	< 0.001
HAS-BLED score, mean (SD)	1.4 (0.9)	1.2 (0.7)	2.3 (0.8)	< 0.001
Comorbidity				
Congestive heart failure, *n* (%)	1864 (26.1)	1526 (27.5)	338 (21.2)	< 0.001
Hypertension, *n* (%)	5065 (70.9)	3920 (70.7)	1145 (71.8)	0.405
Diabetes mellitus, *n* (%)	1737 (24.3)	1312 (23.7)	425 (26.6)	0.017
Angina pectoris, *n* (%)	833 (11.7)	627 (11.3)	206 (12.9)	0.08
Peripheral arterial disease, *n* (%)	187 (2.6)	130 (2.3)	57 (3.6)	0.007
Deep vein thrombosis, *n* (%)	37 (0.5)	24 (0.4)	13 (0.8)	0.061
Pulmonary embolism, *n* (%)	18 (0.3)	15 (0.3)	3 (0.2)	0.562
History of myocardial infarction, *n* (%)	298 (4.2)	223 (4.0)	75 (4.7)	0.247
Aortic aneurysm, *n* (%)	98 (1.4)	73 (1.3)	25 (1.6)	0.45
Dyslipidemia, *n* (%)	2995 (41.9)	2292 (41.3)	703 (44.1)	0.048
Liver dysfunction, *n* (%)	413 (5.8)	339 (6.1)	74 (4.6)	0.023
Medical history				
Stroke (ischemic/hemorrhagic),* n* (%)	1529 (21.4)	86 (1.6)	1443 (90.5)	< 0.001
Ischemic stroke,* n* (%)	1440 (20.2)	0 (0.0)	1440 (90.3)	–
Hemorrhagic stroke, * n* (%)	135 (1.9)	86 (1.6)	49 (3.1)	< 0.001
TIA, *n* (%)	219 (3.1)	0 (0.0)	219 (13.7)	–
SE, *n* (%)	59 (0.8)	38 (0.7)	21 (1.3)	0.018
Malignant tumor, *n* (%)	654 (9.2)	499 (9.0)	155 (9.7)	0.316
Bleeding and/or bleeding tendency, *n* (%)	292 (4.1)	199 (3.6)	93 (5.8)	< 0.001
Dose of rivaroxaban 15 mg/day, *n* (%)	4036 (56.5)	3171 (57.2)	865 (54.2)	0.038
Type of AF (non-paroxysmal (persistent/permanent)), *n* (%)	3940 (55.2)	3022 (54.5)	918 (57.6)	0.031
Prior warfarin use, *n* (%)	2834 (39.7)	2105 (38.0)	729 (45.7)	–
Use of concomitant anti-platelet drugs,* n *(%)	1029 (14.4)	678 (12.2)	351 (22.0)	< 0.001

SD standard deviation, TIA transient ischemic attack, SE systemic embolism, AF atrial fibrillation

There were 1345 (24.3%) new users and 4201 (75.7%) current users in the primary prevention group, and 395 (24.8%) new users and 1200 (75.2%) current users in the secondary prevention group.

### Efficacy

**Fig. 1:**
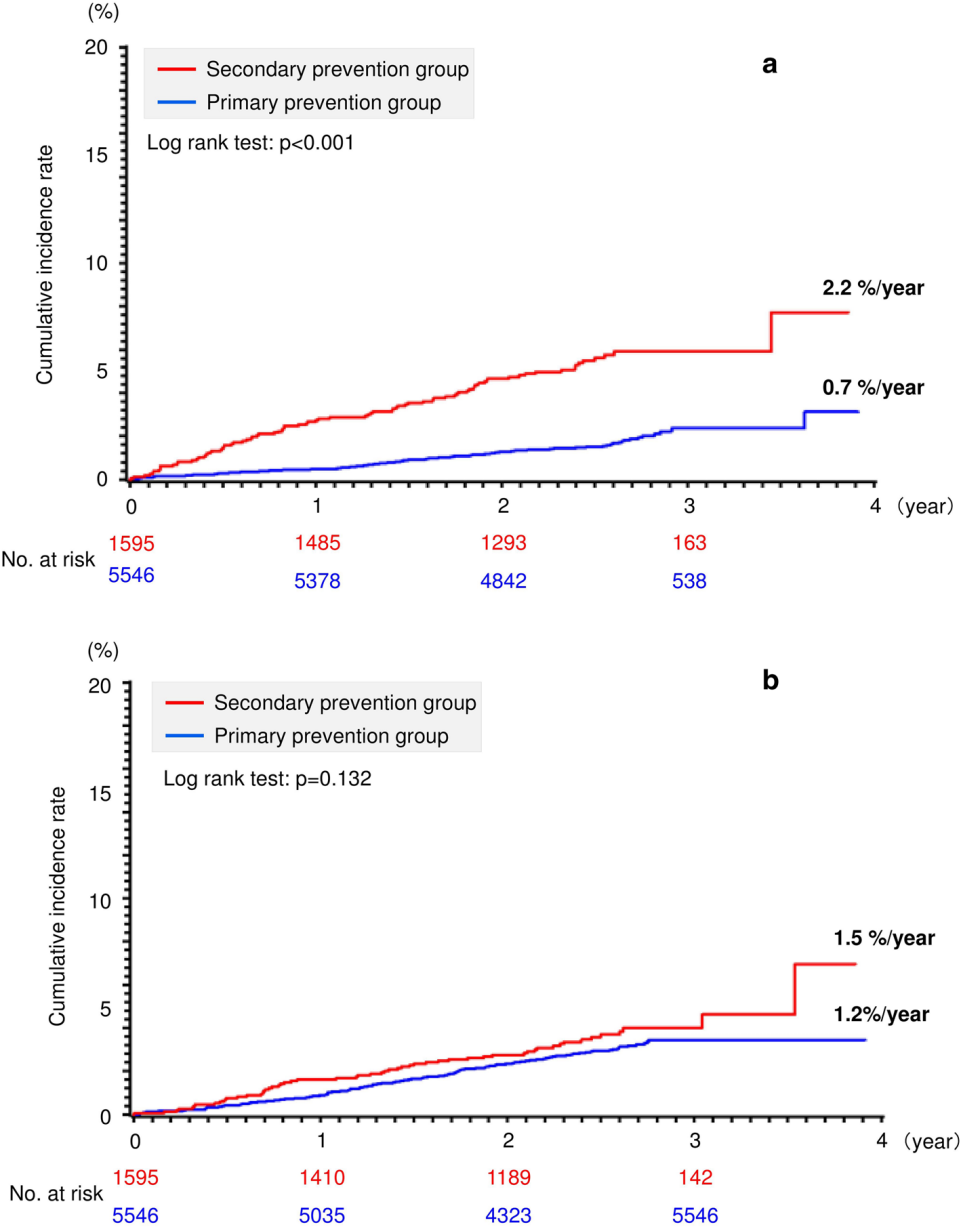
Kaplan–Meier analysis for the primary **a** efficacy and **b** safety endpoints according to primary and secondary prevention groups (efficacy and safety populations)

Stroke (ischemic or hemorrhagic) and SE occurred in 92 patients (0.7%/year) and 84 patients (2.2%/year) in the primary and secondary prevention groups, respectively (*P *< 0.001) (Table 2, Fig. 1a). Among the secondary efficacy endpoints, the following events were significantly more common in the secondary prevention group compared with the primary prevention group; composite of stroke/SE/myocardial infarction (MI)/cardiovascular death, stroke (ischemic/hemorrhagic), ischemic stroke, TIA, SE, cardiovascular death, and all-cause death (Table 2).

**Table 2:** Efficacy and safety endpoints in the primary and secondary prevention groups for stroke and systemic embolism (efficacy and safety populations)

	Primary prevention group (n = 5546)			Secondary prevention group (n = 1595)			P value
	Event, n	%/year	95% CI	Event, n	%/year	95% CI	
Primary efficacy endpoint							
Stroke (ischemic/hemorrhagic)/SE	92	0.7	0.53–0.81	84	2.2	1.72–2.65	< 0.001
Secondary efficacy endpoints							
Stroke (ischemic/hemorrhagic)/SE/MI/cardiovascular death	173	1.3	1.08–1.45	118	3.1	2.51–3.62	< 0.001
Stroke (ischemic/hemorrhagic)	91	0.7	0.53–0.80	80	2.1	1.62–2.54	< 0.001
Ischemic stroke	62	0.5	0.34–0.57	68	1.8	1.35–2.19	< 0.001
Hemorrhagic stroke	28	0.2	0.13–0.28	12	0.3	0.14–0.49	0.21
TIA	18	0.1	0.07–0.19	19	0.5	0.27–0.72	< 0.001
SE	2	0.01	0.00–0.03	4	0.1	0.00–0.21	0.008
Acute MI/unstable angina pectoris	25	0.2	0.11–0.25	10	0.3	0.10–0.42	0.339
Deep vein thrombosis/pulmonary thromboembolism	4	0.03	0.00–0.06	2	0.05	0.00–0.12	0.497
PCI/CABG	30	0.2	0.14–0.30	14	0.4	0.17–0.55	0.109
Cardiovascular death	88	0.6	0.51–0.78	39	1.0	0.70–1.33	0.016
All-cause death	190	1.4	1.19–1.58	91	2.4	1.88–2.85	< 0.001
Primary safety endpoint							
ISTH major bleeding	159	1.2	0.98–1.34	56	1.5	1.70–1.84	0.132
Intracranial hemorrhage	54	0.4	0.29–0.50	30	0.8	0.50–1.06	0.002
Gastrointestinal bleeding	67	0.5	0.37–0.61	16	0.4	0.21–0.62	0.42
Others	38	0.3	0.19–0.37	10	0.3	0.10–0.42	0.942
Secondary safety endpoints							
Non-major bleeding (not defined using ISTH criteria)	668	4.9	4.51–5.25	188	4.9	4.19–5.59	0.963

CI confidence interval, SE systemic embolism, MI myocardial infarction, TIA transient ischemic attack, PCI percutaneous coronary intervention, CABG coronary artery bypass graft, ISTH International Society on Thrombosis and Haemostasis

In the primary and secondary prevention groups, the incidence rate of stroke/SE had no difference between new and current users (primary prevention group: 0.8 vs. 0.6%/year; *P *= 0.271, secondary prevention group: 2.2 vs. 2.2%/year; *P *= 0.959) (Table 3).

**Table 3:** Efficacy and safety endpoints in new and current rivaroxaban users according to primary and secondary prevention groups (efficacy and safety populations)

No. of patient	Primary prevention group		*P* value	Secondary prevention group		*P* value
	New users (*n* = 1345)	Current users (*n* = 4201)		New users (*n* = 395)	Current users (*n* = 1200)	
	Event,* n* (%/year)	Event,* n* (%/year)		Event,* n* (%/year)	Event,* n* (%/year)	
Efficacy endpoints						
Stroke/SE	23 (0.8)	69 (0.6)	0.271	19 (2.2)	65 (2.2)	0.959
All-cause death	54 (1.8)	136 (1.3)	0.003	29 (3.4)	62 (2.1)	0.014
Safety endpoints						
ISTH major bleeding	50 (1.7)	109 (1.0)	0.003	15 (1.8)	41 (1.4)	0.373
Intracranial bleeding	15 (0.5)	39 (0.4)	0.181	8 (0.9)	22 (0.7)	0.604
Gastrointestinal bleeding	22 (0.8)	45 (0.4)	0.051	5 (0.6)	11 (0.4)	0.434
Others	13 (0.4)	25 (0.2)	0.047	2 (0.2)	8 (0.3)	0.979
Non-major bleeding	176 (6.0)	492 (4.6)	0.002	48 (5.6)	140 (4.7)	0.346

SE systemic embolism, ISTH International Society on Thrombosis and Haemostasis

### Safety

ISTH major bleeding events occurred in 159 patients (1.2%/year) and 56 patients (1.5%/year) in the primary and secondary prevention groups, respectively (*P *= 0.132) (Table 2, Fig. 1b). Among the ISTH major bleeding events, the incidence rate of intracranial hemorrhage had significant difference between the primary and secondary prevention groups (0.4 and 0.8%/year, *P* = 0.002). The incidence rate of gastrointestinal bleeding did not differ significantly between the primary and secondary prevention groups (0.5 vs. 0.4%/year; *P *= 0.42). The incidence rate of non-major bleeding had no difference between the 2 groups (4.9 and 4.9%/year*, P *= 0.963) (Table 2).

In the primary prevention group, the incidence rate of ISTH major bleeding and non-major bleeding had significant difference between new and current users (ISTH major bleeding: 1.7 vs. 1.0%/year; *P *= 0.003, non-major bleeding: 6.0 vs. 4.6%/year; *P *= 0.002), while those rate had no difference between the 2 groups in the secondary prevention group (ISTH major bleeding: 1.8 vs. 1.4%/year; *P *= 0.373, non-major bleeding: 5.6 vs. 4.7%/year; *P *= 0.346) (Table 3).

## Discussion

This was a sub-analysis report by the preventive group for stroke or SE in the EXPAND Study to observe the efficacy and safety of Japan-specific dosages of rivaroxaban in Japanese patients with NVAF in a real-world clinical setting. This report provides the first evidence on the efficacy and safety of Japan-specific dosage of rivaroxaban in Japanese patients in a real-world clinical setting according to primary and secondary prevention.

Similar to the previous reports of the sub-analyses for the phase III trials of rivaroxaban (ROCKET and J-ROCKET AF Trial conducted globally and in Japan, respectively) [12, 13], the EXPAND Study showed that the incidence of stroke or SE was higher in the secondary prevention group compared with the primary prevention group. In the EXPAND Study, cardiovascular death and all-cause death were significantly higher in the secondary prevention group compared with the primary prevention group. There were no differences in the incidences of cardiovascular events, including acute MI, unstable angina pectoris, percutaneous coronary intervention/coronary artery bypass graft, or the incidence of ISTH major bleeding events. The incidence of intracranial hemorrhage was higher in the secondary prevention group compared with the primary prevention group among the sites of ISTH major bleeding events. Thus, as compared with the previous sub-analysis reports [12, 13], the EXPAND Study showed similar outcomes except for the low incidence rate of all ISTH major bleeding events.

The demographic characteristics of the patients enrolled in the EXPAND Study and the J-RHYTHM Registry were similar, demonstrating the real-world practice [10, 11, 14, 15]. The mean CHADS_2_ score was 1.6 and 1.4 points for the primary prevention group, and 3.7 and 3.4 points for the secondary prevention group in the EXPAND Study and J-RHYTHM Registry, respectively. Regarding the incidences of events, both the EXPAND Study and the J-RHYTHM Registry showed higher incidence of thromboembolism (ischemic stroke/TIA/SE) in the secondary prevention group [14]. Meanwhile, for ISTH major bleeding events, only the J-RHYTHM Registry showed a different incidence between primary and secondary prevention groups (primary 1.7% vs. secondary 3.0%, *P *= 0.003). This higher incidence of bleeding was likely to arise through the higher combined usage of warfarin and antiplatelet agents in the secondary prevention group compared with the primary prevention group in the J-RHYTHM Registry (primary 16.1% vs. secondary 32.4%, *P *< 0.001). The EXPAND Study also showed higher combined use of antiplatelet agents in the secondary prevention group (12.2 vs. 22.0%, *P *< 0.001). Moreover, the HAS-BLED score in the EXPAND Study was higher in the secondary prevention group than in the primary prevention group (1.2 vs. 2.3 points, *P *< 0.001) (Table 1). However, the incidence rates of ISTH major and no-major bleeding were comparable between the primary and secondary prevention groups (ISTH major bleeding; 1.2 vs 1.5%/year, *P *= 0.132) (non-major bleeding; 4.9 vs. 4.9%/year, *P *= 0.963) (Table 2). However, no difference was noted in the incidence of major bleeding events between the primary and secondary prevention groups (1.2 vs. 1.5%/year, *P *= 0.132). The patients who treated with concomitant use of warfarin and aspirin had higher risk of bleeding compared with those who treated with warfarin alone [16]. Other reports have also described increased incidences of bleeding events with combined use of anticoagulant and antiplatelet drugs [17–19]. Further studies on the risk of developing bleeding events under combined use of rivaroxaban and antiplatelet drugs in comparison with combination of warfarin and antiplatelet drugs should be conducted.

In the sub-analysis for the phase III trials of rivaroxaban (ROCKET and J-ROCKET AF Trials), no significant differences were noted for efficacy (stroke/SE) or safety (ISTH major bleeding) between the primary and secondary prevention groups treated with rivaroxaban and warfarin [12, 13]. However, in both warfarin and rivaroxaban groups, the secondary prevention group had higher incidences of stroke and SE compared with the primary prevention group, while the primary prevention group had a higher incidence of major bleeding events compared with the secondary prevention group [12, 13] (Table 4). In those trials, the primary prevention group had more risk factors of bleeding as components of the HAS-BLED score [20] than the secondary prevention group (Table 5). It is possible that the primary prevention group had higher risk of bleeding compared with the secondary prevention group in general.

**Table 4:** Comparisons of primary and secondary prevention groups in two previous clinical trials and the EXPAND Study

(A) Demographic and baseline clinical characteristics of patients		
	Mean CHADS_2_ score	
	Primary prevention group	Secondary prevention group
ROCKET AF Trial	3	4
J-ROCKET AF Trial	2.9	3.5
EXPAND Study	1.6	3.7

(B) Primary efficacy and safety endpoints				
	Primary efficacy endpoint (stroke/systemic embolism)		Primary safety endpoints (major bleeding)	
	Primary prevention group	Secondary prevention group	Primary prevention group	Secondary prevention group
ROCKET AF Trial (%/year)	1.4	2.8	4.1	3.1
J-ROCKET AF Trial (%/year)	0.6	1.7	4.0	2.4
EXPAND Study (%/year)	0.7	2.2	1.2	1.5

**Table 5:** Comparisons of two previous clinical trials and the EXPAND Study according to the risk factor of bleeding as component of the HAS-BLED score

	ROCKET AF Trial			J-ROCKET AF Trial			EXPAND Study		
	Primary prevention group	Secondary prevention group	*P* value	Primary prevention group	Secondary prevention group	*P* value	Primary prevention group	Secondary prevention group	*P* value
Hypertension, (%)	96	85	< 0.001	95.7	70.3	–	70.7	71.8	0.405
Mean CrCl, (ml/min)	65	69	< 0.001	67.1	68.1	–	71.2	64.7	< 0.001
CrCl < 50 ml/min, (%)	–	–	–	26.4	19.6	–	20.3	26.8	< 0.001
Mean age, (years)	75	71	< 0.001	72.2	70.3	–	70.9	73.9	< 0.001
Antiplatelet use (aspirin), (%)	35	38	0.004	35.5	39.5	–	9.1	11.6	–

CrCl creatinine clearance

In the EXPAND Study, the incidences of stroke and SE were similar to those in the studies mentioned above. However, regarding the incidence of major and non-major bleeding events, no significant difference was noted between the primary and secondary prevention groups in this study (Table 2). The possible reasons are that the ROCKET and J-ROCKET AF Trials did not include patients with CHADS_2_ scores of 0 and 1 [8, 9], while the EXPAND Study did include those with such scores [10, 11], and that the ROCKET and J-ROCKET AF Trial potentially had more patients with risk of bleeding in the primary prevention group than the EXPAND Study (Table 4). In addition to that, the differences in CHADS_2_ and HAS-BLED scores were approximately two and one scores between the 2 groups of those scores in the EXPAND Study, respectively. Consequently, the patient background of each prevention group in this study did not differ significantly except for the history of stroke. As shown in Table 4, the incidence rate of stroke or SE in the secondary prevention group of the EXPAND Study was higher than that of the J-ROCKET AF Trial (2.2 and 1.7%/year, respectively), even though mean CHADS_2_ score in the EXPAND Study was lower than that in the J-ROCKET AF Trial (1.6 and 2.9 points, respectively). By contrast, the incidence rate of ISTH major bleeding in both the primary and secondary prevention groups of the EXPAND Study were much lower (1.2 and 1.5%/year, respectively) than those of the J-ROCKET AF Trial (4.0 and 2.4/year, respectively), despite mean CHADS_2_ scores of the secondary prevention group were comparable between the EXPAND Study and J-ROCKET AF Trial (3.7 and 3.5 points, respectively). We consider that off-label dose reduction was attributable to these results. Furthermore, it could be another reason for the higher incidence rate of stroke or SE in the secondary prevention group of the EXPAND Study that patients with acute stage of stroke within 2 weeks after the onset, who were at high risk of recurrence, were included in the EXPAND Study but not in the J-ROCKET AF Trial. We plan to clarify these issues in the ongoing exploratory analyses of our study.

In the EXPAND Study, the incidence of intracranial hemorrhage was higher in the secondary prevention group than in the primary prevention group (primary 0.4%/year vs. secondary 0.8%/year, *P *=0.002), but gastrointestinal bleeding rate was not different between the 2 groups (primary 0.5%/year vs. secondary 0.4%/year, *P *=0.42). These results suggest that history of stroke is a risk factor of intracranial hemorrhage, but not a risk factor of gastrointestinal bleeding. The incidence of intracranial hemorrhage was higher in the secondary prevention group and the warfarin-treated group compared with the primary prevention group in the J-ROCKET AF Trial [9] as well. Further studies are needed to evaluate background factors with potential impacts on bleeding events and sites in the primary and secondary prevention groups.

### Study limitations

As already reported [11], the study has several limitations. In the sub-analysis as well, information bias may have had an effect on the results. Current users may not include subjects developing adverse events such as bleeding after prescription. In the sub-analysis, the incidence of major bleeding events in the primary prevention group was significantly higher in new users compared with current users. Although the difference was not significant, probably because of the difference in sample size, the incidence of major bleeding events was still higher in current users than in new users. Moreover, all-cause mortality in new users was significantly higher in both the primary and secondary prevention groups, suggesting another impact of information bias. By contrast, for the primary efficacy endpoint, the findings are considered reliable because of less selection bias.

## Conclusions

The results of this sub-analysis allowed us to verify the low incidence of primary efficacy and safety endpoints in patients on Japan-specific dosages of rivaroxaban for not only primary but also secondary prevention of stroke and SE in Japanese patients with NVAF in a real-world clinical setting. In addition, as reported previously, the incidences of stroke and SE were higher in the secondary prevention group than in the primary prevention group. For bleeding events, further studies are needed to evaluate the difference in bleeding sites as our results were not consistent with those in the previous reports.

## Electronic supplementary material

Below is the link to the electronic supplementary material.

Supplementary material 1 (DOCX 30 kb)

## Abbreviations

AF: Atrial fibrillation
CABG: Coronary artery bypass grafting
CI: Confidence interval
CrCl: Creatinine clearance
ISTH: International Society on Thrombosis and Haemostasis
MI: Myocardial infraction
NOAC: Non-vitamin K antagonist oral anticoagulant
NVAF: Non-valvular atrial fibrillation
PCI: Percutaneous coronary intervention
SD: Standard deviation
SE: Systemic embolism
TIA: Transient ischemic attack
