# Supplementary material for: Primary and secondary prevention of stroke and systemic embolism with rivaroxaban in patients with non-valvular atrial fibrillation: Sub-analysis of the EXPAND Study
Source: Heart Vessels. 2018 Jul 6;34(1):141–50. doi: 10.1007/s00380-018-1219-0 (PMC6347661; doi:10.1007/s00380-018-1219-0)
Supplement: Supplementary file 1 — Supplementary material 1 (DOCX 30 kb) [file 380_2018_1219_MOESM1_ESM.docx]

**Supplementary material**

**Study organisation**

List of affiliations as of August 2016.

1. **Steering committee**

Hiroaki Shimokawa (Department of Cardiovascular Medicine, Tohoku University Graduate School of Medicine)

Hirotsugu Atarashi (Tsurumaki Onsen Hospital)

Hiroshi Inoue (Saiseikai Toyama Hospital)

Shinichiro Uchiyama (Clinical Research Center for Medicine, International University of Health and Welfare)

Takanari Kitazono (Department of Medicine and Clinical Science, Graduate School of Medical Sciences, Kyushu University)

Takeshi Yamashita (Cardiovascular Institute Hospital)

Wataru Shimizu (Department of Cardiovascular Medicine, Graduate School of Medicine, Nippon Medical School)

1. **Protocol committee**

Takanori Ikeda (Department of Cardiovascular Medicine, Toho University Faculty of Medicine)

Koichi Kaikita (Department of Cardiovascular Medicine, Kumamoto University)

Masahiro Kamouchi (Department of Health Care Administration and Management, Kyushu University Graduate School of Medical Sciences)

Koji Fukuda (Department of Cardiovascular Medicine, Tohoku University Graduate School of Medicine)

1. **Clinical event committee**

[Cardiac Region]

Kurita Takashi (Division of Cardiology, Department of Medicine, Faculty of Medicine, Kindai University)

Ken Kozuma (Department of Cardiology, Teikyo University Hospital)

Kazuhiko Nishigaki (Department of Cardiology, Respirology, and Nephrology, Gifu University Graduate School of Medicine)

[Brain Region]

Kazutoshi Nishiyama (Department of Neurology, Kitasato University School of Medicine)

Teruyuki Hirano (Department of Neurosurgery, Kyorin University Faculty of Medicine)

Haruhiko Hoshino (Department of Internal Medicine, Department of Neurology, Tokyo Saiseikai Central Hospital)

1. **Principal statistician**

Hideki Origasa (Department of Biostatistics and Clinical Epidemiology, Toyama University)

1. **Enrolment promotion committee**

[Cardiac Region]

Hiroyuki Tsutsui (Department of Cardiovascular Medicine, Hokkaido University Graduate School of Medicine)

Naoyuki Hasebe (Department of Cardiology, Pulmonology, and Nephrology, Asahikawa Medical University Hospital)

Isao Kubota (Department of Cardiology, Pulmonology, and Nephrology, Yamagata University Faculty of Medicine)

Hiroshi Ito (Division of Cardiovascular and Respiratory Medicine, Department of Internal Medicine, Akita University Graduate School of Medicine)

Hiroyuki Daida (Department of Cardiovascular Medicine, Juntendo University Graduate School of Medicine)

Atsushi Hirayama (Department of Cardiovascular Medicine, Nihon University School of Medicine)

Nobuhisa Hagiwara (Department of Cardiovascular Medicine, Tokyo Women's Medical University)

Kazuo Kimura (Cardiovascular Center, Yokohama City University Medical Center)

Yoshio Kobayashi (Department of Cardiovascular Medicine, Chiba University Graduate School of Medicine)

Shinichi Momomura (Division of Cardiology, Saitama Medical Center Jichi Medical University)

Kazuomi Kario (Division of Cardiovascular Medicine, Department of Medicine, Jichi Medical University of Medicine)

Kazutaka Aonuma (Cardiovascular Division, University of Tsukuba Faculty of Medicine)

Teruo Inoue (Department of Cardiovascular Medicine, Dokkyo Medical University)

Uichi Ikeda (Department of Cardiovascular Medicine, Shinshu University School of Medicine)

Tohru Minamino (Department of Cardiovascular Biology and Medicine, Niigata University Graduate School of Medical and Dental Sciences)

Toyoaki Murohara (Department of Internal Medicine, Nagoya University Graduate School of Medicine)

Shinya Minatoguchi (Department of Cardiology, Respirology, and Nephrology, Gifu University Graduate School of Medicine)

Masakazu Yamagishi (Department of Cardiology, Kanazawa University Hospital)

Issei Komuro (Department of Cardiovascular Medicine, Tokyo University Graduate School of Medicine)

Satoshi Yasuda (Department of Cardiovascular Medicine, National Cerebral and Cardiovascular Center)

Ken-ichi Hirata (Division of Cardiovascular Medicine, Department of Internal Medicine, Kobe University Graduate School of Medicine)

Tohru Masuyama (Cardiovascular Division, Department of Internal Medicine, Hyogo College of Medicine)

Minoru Horie (Department of Cardiovascular and Respiratory Medicine, Shiga University of Medical Science Hospital)

Takashi Akasaka (Division of Cardiovascular Medicine, Wakayama Medical University)

Yasuki Kihara (Department of Cardiovascular Medicine, Hiroshima University Graduate School of Biomedical and Health Sciences)

Hiroshi Ito (Department of Cardiovascular Medicine, Okayama University Graduate School of Medicine, Dentistry and Pharmaceutical Sciences)

Kazuaki Tanabe (Fourth Department of Internal Medicine, Shimane University Faculty of Medicine)

Jitsuo Higaki (Department of Cardiology, Pulmonology, Hypertension & Nephrology, Ehime University Graduate School of Medicine)

Shigenobu Bando (Director, Kagawa Prefectural Shirotori Hospital)

Koji Maemura (Department of Cardiovascular Medicine, Nagasaki University Hospital)

Keijiro Saku (Department of Cardiology, Fukuoka University Faculty of Medicine)

Yoshihiro Fukumoto (Division of Cardiovascular Medicine, Department of Internal Medicine, Kurume University School of Medicine)

Koichi Node (Department of Cardiovascular Medicine, Saga University Faculty of Medicine)

Shinichiro Ueda (Department of Clinical Pharmacology & Therapeutics, University of the Ryukyus Graduate School of Medicine)

Yusuke Ohya (Department of Cardiovascular Medicine, Nephrology, and Neurology, University of the Ryukyus Graduate School of Medicine)

[Brain Region]

Kiyohiro Houkin (Department of Neurosurgery, Hokkaido University Graduate School of Medicine)

Ken Nagata (Department of Neurology, Research Institute for Brain and Blood Vessels-Akita)

Eisuke Furui (Departments of Stroke Neurology, Kohnan Hospital)

Koichi Hirata (Department of Neurology, Dokkyo Medical University)

Ban Mihara (Department of Neurology, Mihara Memorial Hospital)

Norio Tanahashi (Department of Neurology, Saitama Medical University International Medical Center)

Shiro Kobayashi (Department of Neurosurgery, Nippon Medical School Chiba Hokusou Hospital)

Norihiro Suzuki (Department of Neurology, Keio University School of Medicine)

Yasuo Katayama (Department of Neurological Sciences, Nippon Medical School Graduate School of Medicine)

Shunya Takizawa (Division of Neurology, Department of Internal Medicine, Tokai University School of Medicine)

Yasuhiro Hasegawa (Division of Neurology, Department of Internal Medicine, St. Marianna University School of Medicine)

Kazuo Yamada (Department of Neurosurgery, Nagoya City University Graduate School of Medical Sciences)

Gen Sobue (Department of Neurology, Nagoya University Graduate School of Medicine)

Kotaro Tanaka (Department of Neurology, Toyama University Hospital)

Susumu Miyamoto (Department of Neurosurgery, Kyoto University Graduate School of Medicine)

Yasumasa Yamamoto (Department of Neurology, Kyoto Second Red Cross Hospital)

Kazunori Toyoda (Department of Cerebrovascular Medicine, National Cerebral and Cardiovascular Center)

Kazuyuki Nagatsuka (Department of Neurology, National Cerebral and Cardiovascular Center)

Kazuo Kitagawa (Department of Neurology, Osaka University Graduate School of Medicine)

Masayasu Matsumoto (Department of Clinical Neuroscience & Therapeutics, Hiroshima University Graduate School of Biomedical and Health Sciences)

Koji Abe (Department of Neurology, Okayama University Graduate School of Medicine, Dentistry and Pharmaceutical Sciences)

Kazumi Kimura (Department of Stroke Medicine, Kawasaki Medical School)

Shinji Nagahiro (Department of Neurosurgery, Institute of Health Biosciences, Tokushima University Graduate School of Medicine)

Yasushi Okada (Clinical Research Institute, National Hospital Organization Kyushu Medical Center)

Yoichiro Hashimoto (Department of Neurology, Kumamoto City Hospital)

Izumi Nagata (Department of Neurosurgery, Nagasaki University Hospital)

1. **Publication committee**

The publication committee consisted of the steering committee members.

1. **Research administration office**

The research administration office was set up in Mebix, Inc. (1-11-44 Akasaka, Minato-ku, Tokyo, Japan)

Chief Officer: Yasuhiko Yoshida

Administration Office Manager: Koji Fukuda (Department of Cardiovascular Medicine, Tohoku University Graduate School of Medicine)

Administration Office Assistant Manager: Eiko Ishida (Department of Cardiovascular Medicine, Tohoku University Graduate School of Medicine)

1. **Data centre**

The data centre was set up in Mebix, Inc. (1-11-44 Akasaka, Minato-ku, Tokyo, Japan)

Chief Officer: Yukio Yamada

1. **Monitoring organisation**

Mebix, Inc. (1-11-44 Akasaka, Minato-ku, Tokyo, Japan)

Chief Officer: Yasuhiko Yoshida

Mediscience Planning Inc. (1-11-44 Akasaka, Minato-ku, Tokyo, Japan)

MIC Medical Corp. (1-11-44 Akasaka, Minato-ku, Tokyo, Japan)
